# Spontaneous renal pelvis haematoma presenting as renal colic

**DOI:** 10.1259/bjrcr.20180086

**Published:** 2018-10-02

**Authors:** Yakup Kilic, Yunus Gokdogan, Sharif Abdullah

**Affiliations:** 1 Department of Emergency Medicine, Westmiddlesex Hospital, Isleworth, UK

## Abstract

Spontaneous renal pelvic haematomas are rare, often mimicking symptoms of other pathologies such as a renal tumour or renal calculi. Spontaneous renal haematoma was first reported by Bonet in 1679 and later described by Wunderlich in 1856. We present the case of a young female patient with no known comorbidities who presented with spontaneous renal pelvis haematoma. Misinterpretation of this finding can lead to erroneous diagnoses.

## Case presentation

A fit and well 39-year-old female self-presented to the emergency department with a 2-day history of right-sided flank pain radiating to the back, colicky in nature. This was associated with frank haematuria. The medical history and examination were unremarkable with no known comorbidities. Furthermore, the patient was not on any anti coagulants or anti platelets and there was no history of trauma.

## Investigation

A CT-KUB was performed. This demonstrates a 2.2 × 2.4 cm branching high attenuation opacity in the right renal pelvis. Mild right perinephric stranding with no urinary tract calculi or ureteric dilatation was noted. Appearances are consistent with a right renal pelvis haematoma. Comparison was not available since the patient had no previous CTs. The eGFR was 67, otherwise blood results were unremarkable with no decrease in haemoglobin. The patient was then admitted under the urology team for further observations and care.

## Treatment

The initial treatment for the patient in our A&E department consisted of symptomatic management. This involved administering 1g Paracetamol, 20 mg Buscopan, 100 mg Diclofenac PR and 10 mg of Morphine. Pain was resistant to simple analgesics and only responded to PR and i.v. medication. Fluid resuscitation with Plasmalyte and Ondansetron were also administered. Furthermore, i.v. Ciprofloxacin was prescribed as prophylactic measure.

## Differential diagnoses

The most common diagnosis in a young patient presenting with severe flank pain in the absence of fever is renal colic. Other differential diagnoses for this CT appearance would include haemorrhagic transitional cell carcinoma and hyperdense parapelvic cyst. On non-contrast CT, TCC lesions are of soft tissue attenuation between 20–70 HU.^1,2^ Larger lesions frequently have foci of necrosis. Approximately 30% show some calcification.^3^ An abscess would appear on CT as a well-defined mass of low attenuation with a thick, irregular wall or pseudo capsule (reference). Renal parenchyma around the abscess cavity may appear hypoenhancing in the nephrogram phase and may appear hyperattenuating on delayed images. Renal cysts depending on the Bosniak classification can appear as well-defined with a thin wall and hyperattenuating if greater than 20 HU.^1,2^

## Outcome and follow up

As the patient was experiencing ongoing haematuria, she underwent a CT-IVU and flexible cystoscopy as an outpatient. The CT-IVU showed resolution of the right renal pelvic haematoma with no evidence of hydronephrosis or hydroureter. Flexible cystoscopy demonstrated mild trabeculation and some mild inflammation around the trigone and no malignant cells were found on cytopathology. The patient was prescribed prophylactic Trimethoprim and was discharged with specialist nurse follow-up in the lower urinary tract symptoms clinic.

## Discussion

Renal pelvic haematomas usually present in patients with pre-existing renal pathology such as renal stones, cancer or in the setting of trauma.^4^ Having a non-traumatic spontaneous pelvic haematoma in a patient who is otherwise fit and well is very rare with only a few reported cases in the literature. There have been reported cases of large renal pelvis haematomas in uretero-pelvic junction obstruction presenting as an acute abdomen.^5^ In comparison, our patient did not have an acute abdomen, rather only colicky right-sided flank pain.

Thorough investigation is necessary to reduce interpretation errors that can lead to erroneous nephrectomy.^6^ This is due to renal haematoma being misinterpreted as a renal mass. Consequently, contrast-enhanced CT or MRI may be considered to exclude other diagnoses. Intrarenal causes may also lead to the symptoms demonstrated by this patient. For example, spontaneous renal pelvic haematomas have also been found mimicking cancer in IgA nephropathy.^6^ This occurs due to deposition of pathogenic immune complexes in the mesangium leading to glomerular injury.^7^ However, in our case no obvious pathogenesis can explain how the haematoma formed and resolved in a short period of time. Consequently, having more literature comparing renal pelvis haematomas mimicking other pathologies may help radiologists make more definitive decisions when reporting. Lastly, the majority of cases reported are of right renal pelvic haematomas. The reason for this is not understood. Possible explanations are anatomical and/or embryological developmental differences between the right and left kidneys.

## Learning points

1. The wide clinical differential diagnoses of unilateral colicky loin pain should be considered in all patients presenting to emergency departments.
2. This case report highlights the importance of considering all differential diagnoses when reporting CTs demonstrating renal masses. Correlation with the patient’s clinical presentation is key.
3. Contrast can be considered to differentiate between masses and haemorrhage. Contrast can increase CT sensitivity and enhance differentiation among different pathologies.
4. MRI can be considered to aid in diagnosis of renal masses if there is diagnostic uncertainty on CT.
5. CT angiography can be considered for A-V malformations that may lead to renal haematoma.

**Figure 1.**
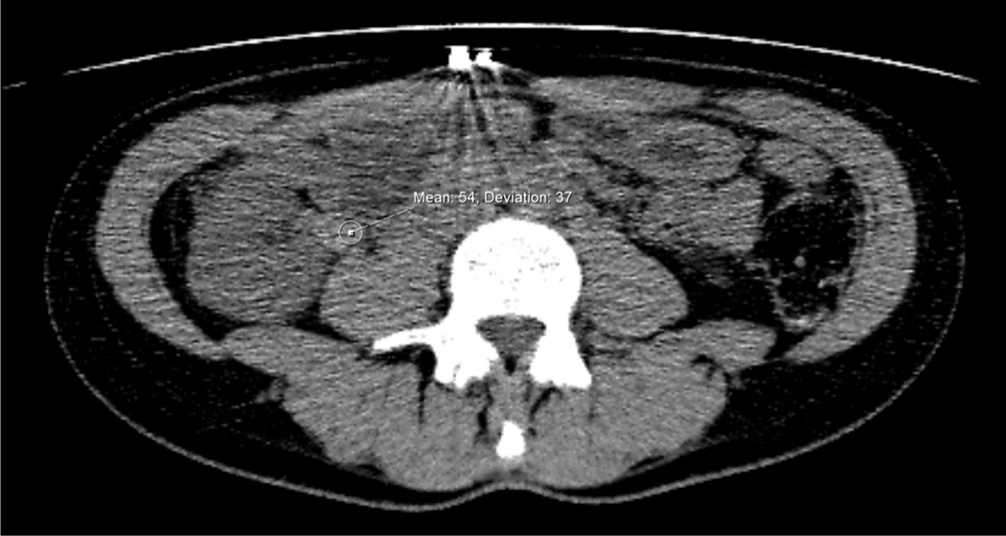
CT-KUB demonstrating a 2.2 × 2.4 cm branching high attenuation opacity in the right renal pelvis.

**Figure 2.**
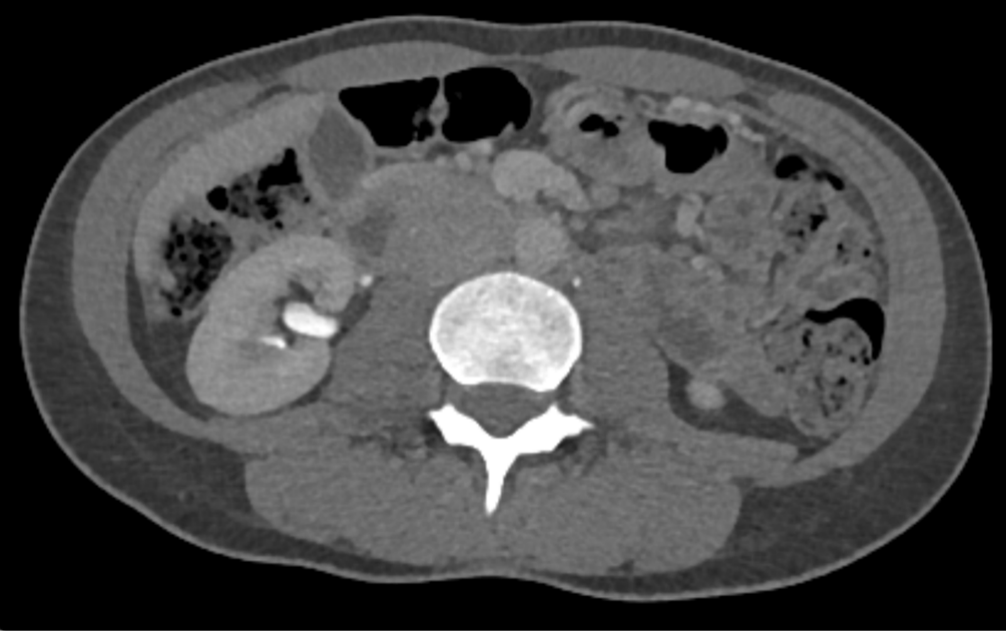
CT-IVU demonstrating resolution of the right renal pelvic haematoma with no evidence of hydronephrosis or hydroureter.

**Figure 3.**
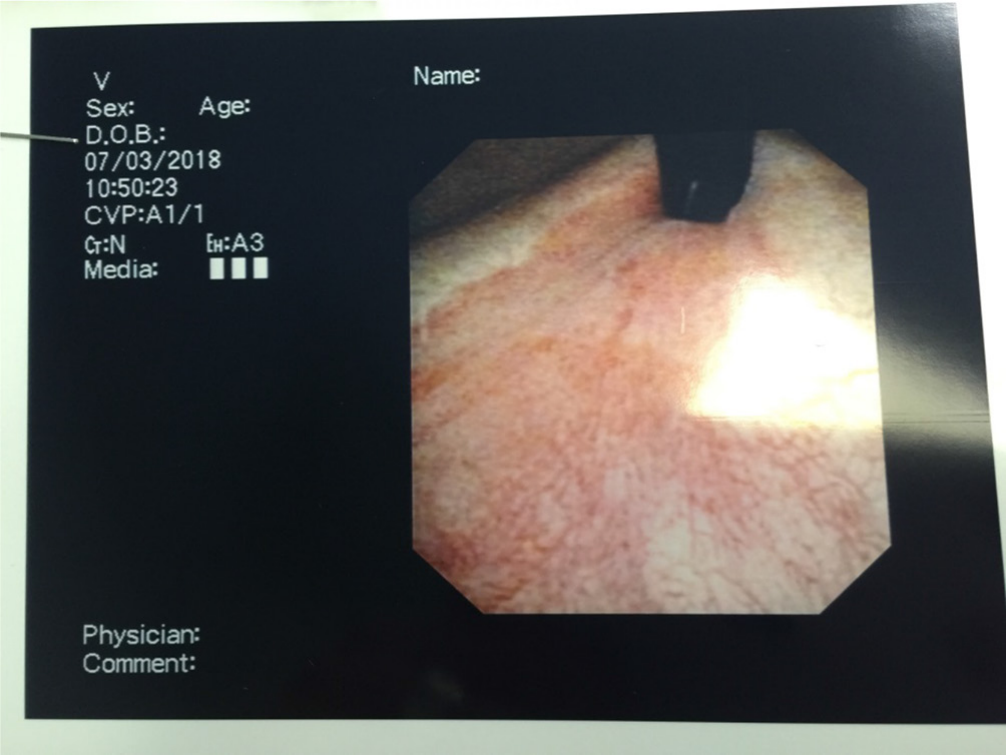
Flexible cystoscopy demonstrating mild trabeculation and some mild inflammation around the trigone.
